# Establishment of a bioluminescent canine B-cell lymphoma xenograft model for monitoring tumor progression and treatment response in preclinical studies

**DOI:** 10.1371/journal.pone.0208147

**Published:** 2018-12-28

**Authors:** Joana N. R. Dias, Ana S. André, Sandra I. Aguiar, Joana Ministro, Joana Oliveira, Maria C. Peleteiro, Barbara Rütgen, Lurdes Gano, João D. G. Correia, Soraia S. Oliveira, Joao Goncalves, Solange Gil, Luís Tavares, Frederico Aires-da-Silva

**Affiliations:** 1 CIISA – Centro de Investigação Interdisciplinar em Sanidade Animal, Faculdade de Medicina Veterinária, Universidade de Lisboa, Lisbon, Portugal; 2 Research Institute for Medicines (iMed.ULisboa), Faculty of Pharmacy, Universidade de Lisboa, Lisbon, Portugal; 3 Department of Pathobiology, Clinical Pathology Unit, University of Veterinary Medicine, Vienna, Austria; 4 Centro de Ciências e Tecnologias Nucleares, Instituto Superior Técnico, Universidade de Lisboa, CTN, Bobadela LRS, Portugal; Cornell University, UNITED STATES

## Abstract

Canine diffuse large B-cell lymphoma (DLBCL) is one of the most common cancers in dogs which shares remarkable similarities with its human counterpart, making the dog an excellent model for the investigation of novel therapeutic agents. However, the integration of canine lymphoma in comparative studies has been limited due in part to the lack of suitable xenograft mouse models for preclinical studies. To overcome these limitations, we established and characterized a localized subcutaneous bioluminescent canine DLBCL xenograft mouse model. The canine CLBL-1 cell line stably expressing the luciferase and green fluorescent protein reporters was generated and used to establish the xenograft tumor model. A pilot study was first conducted with three different cell densities (0.1×10^6^, 0.5×10^6^ and 1×10^6^ cells) in SCID mice. All mice presented homogeneous tumor induction within eight days after subcutaneous injection, with a 100% engraftment efficiency and no significant differences were observed among groups. The tumors were highly aggressive and localized at the site of inoculation and reproduced histological features and immunophenotype consistent with canine DLBCL. Importantly, xenograft tumors were detected and quantified by bioluminescent imaging. To assess response to therapy, a therapeutic study with a histone deacetylase inhibitor, panobinostat, was performed. The results demonstrated that panobinostat (20 mg/kg) efficiently inhibited tumor growth and that bioluminescent imaging allowed the monitorization and quantification of tumor response to therapy. In summary, this study provides a bioluminescence canine DLBCL model that offers high engraftment efficiency, preservation of tumor features, and noninvasive monitoring of tumor progression, validating the model as a promising preclinical tool for both veterinary and human medicine.

## Introduction

Non-Hodgkin lymphoma (NHL) is a leading cause of cancer-related death in the United States and Europe, and its incidence continues to increase [1,2]. NHL encompasses a heterogeneous group of malignancies that usually originates in the lymph nodes, but can occur in almost any tissue, resulting from the neoplastic transformation of B and T lymphocytes [3]. Diffuse large B-cell lymphoma (DLBCL) is the most common subtype of NHL, comprising approximately 30–58% of all NHL cases [2]. DLBCL is an aggressive form of lymphoma that is initially chemoresponsive, showing favorable responses to frontline R-CHOP (rituximab, cyclophosphamide, doxorubicin, vincristine, and prednisolone) immunochemotherapy. However, 10–15% of DLBCL patients are primarily refractory to this treatment and approximately 20–25% relapse after the initial response, with poor survival rates after current salvage therapy regimens [4]. As such, new therapeutic agents and approaches are urgently needed. A multitude of new drugs are entering clinical development for NHL treatment; nevertheless the approval of new therapies remains low due in part to the scarcity of clinically relevant models for validation [5]. Canine DLBCL, one of the most common neoplasia**s** in dogs, shares genetic, biological, molecular and clinical similarities with its human counterpart, making the dog an excellent animal model to explore novel therapeutic molecules and approaches for human DLBCL [6–10]. Moreover, dogs diagnosed with lymphoma are frequently treated with anthracycline based chemotherapy regimens, similarly to human DLBCL patients, providing realistic opportunities to explore therapeutic protocols that may translate to human clinical trials [8]. These initiatives are also encouraged by the increasing healthcare standards demanded by pet owners, creating the need for novel cancer therapies designed for veterinary applications [11–13]. Still, the integration of canine lymphoma as an animal model for clinical validation of therapeutics has been partially limited by the lack of suitable cNHL mouse models for preclinical research [9]. In fact, even though comparative oncology studies provide unique information not easily acquired with conventional preclinical models, the use of the tumor-bearing dog model for innovative drug development requires previous controlled toxicokinetic studies in laboratory animals [14].

Mouse models have been critical tools for studying the biology and genetics of cancer as well as for predicting efficacy and for evaluating toxicity of anti-cancer therapeutics [15,16]. Indeed, the discovery that tumor tissue could be xenografted into T-cell deficient nude athymic (nu/nu) mice [17], and later into B-cell-deficient and T-cell-deficient severe combined immunodeficient (scid/scid) mice [18], started a new era for experimental studies in oncology, allowing the routine and efficient transplantation and propagation of human tumor tissues in mice. In fact, many human xenograft tumor models have been established, especially for human lymphoma, resulting in the identification of therapeutic molecules that continue to lead clinical cancer management as chemotherapy treatments [19].

Despite the increasing investment in canine lymphoma research, there is a paucity of validated, well characterized and widely disseminated canine lymphoma preclinical models. Possibly due to the low number of available well-characterized canine lymphoid cell lines, the majority of *in vivo* canine lymphoma models described represent T-cell lymphoid malignancies [20–22]. Indeed, CLBL-1 cell line is the only canine cell line that faithfully represents diffuse large B-cell lymphoma (DLBCL), reproducibly inducing tumors and preserving its phenotype in the xenotransplantation setting [11,23,24]. Within this context, CLBL-1 xenograft mouse models are the most reliable preclinical tool of canine B-cell lymphoma. Although previous studies [11,24] paved the way for the development of canine B-cell lymphoma mouse models many questions remained to be answered regarding tumor engraftment efficiency, reproducibility and the potential to be used for bioluminescent (BLI) monitoring. Aiming to overcome such limitations, we established and characterized a new localized subcutaneous bioluminescent canine CLBL-1 DLBCL xenograft mouse model using a stable CLBL-1 cell line expressing the luciferase and green fluorescent protein reporters, that easily allows monitoring tumor progression and treatment response in preclinical studies.

## Material and methods

### Cell culture and reagents

The canine CLBL-1 B-cell lymphoma cell line previously established by Dr. Barbara Rütgen, (Department of Pathobiology, University of Veterinary Medicine, Vienna, Austria) [23,24] was cultured in Roswell Park Memorial Institute–1640 (RPMI-1640) medium (Gibco, Life Technologies, Paisley, UK) supplemented with 10% heat inactivated fetal calf serum (FCS, Gibco) and penicillin 100 U/ml plus streptomycin 0.1 mg/ml (Gibco). Cell cultures were maintained at 37°C in a humidified atmosphere of 5% CO_2_ (T75-tissue culture flasks, Greiner Bio-One, Kremsmünster, Austria).

### Construction of a CLBL-1^GFP+luciferase+^ stable cell line

For *in vivo* live imaging, a CLBL-1^GFP+luciferase+^ stable cell line was generated using a lentiviral system encoding firefly luciferase and green fluorescent protein (GFP) reporters. CLBL-1 cells were transduced with luciferase-2A-GFP lentiviral particles (Amsbio Cat#LVP020), according to the manufacturer’s protocol and as previously described [25]. Briefly, 5×10^6^ CLBL-1 cells were resuspended in 100 μl of lentiviral particles (1x10^7^ IFU/ml) and subjected to spinoculation method [26]. After 6h, medium was changed for fresh complete RPMI and after 24h an equal amount of fresh medium was added. At 72h, transduction efficiency was assessed by FACS and GFP positive cells were sorted, using FACSAria IIu sorter (BD Biosciences), and maintained in the same culture medium supplemented with gentamycin 50 μg/ml (Gibco) for 7 days to avoid contamination. After 4 weeks in culture, CLBL-1^GFP+luciferase+^ cells were subjected to a second sort to ensure a stable GFP expressing cell line and cultured as previously described above. Two weeks after the second cell sort and two months following the cell line maintenance, GFP fluorescence was confirmed by FACS analysis. In addition, luciferase activity was confirmed using a luciferase assay kit (Promega, Wisconsin, USA) according to the manufacturer´s protocol. As a control, non-transduced CLBL-1 cells were analyzed in parallel. To confirm that no alteration of cellular physiology occurred during the construction of CLBL-1^GFP+luciferase+^ cell line, we compared growth patterns of both parental and transduced cell lines using a cell doubling time assay as previously described by Rütgen *et al*., 2010 [24]. Finally, CLBL-1^GFP+luciferase+^ cell line was authenticated by short tandem repeat (STR) testing and compared to the parental CLBL-1 cell line (Eurofins Genomics, Ebersberg, Germany).

### Mouse and breeding conditions

All animal-handling procedures were performed according to EU recommendations for good practices and animal welfare, and approved by the Animal Care and Ethical Committee of the Veterinary Medicine Faculty. Female 6–8-wk-old SOPF/SHO SCID mice (Charles River Laboratory) were maintained in microisolation individually ventilated cages under pathogen-free conditions (Tecniplast, Buguggiate, Italy, Boxunsfeu model, with H14 Hepa Filter e Prefilter sheet for Smart Flow). Mice were allowed to acclimatize for at least two weeks prior to the experiment start. Mice were kept on a 12h light: 12h dark cycle. Room temperature was maintained at 24–26°C. Food pellets and water were sterilized and provided *ad libitum*.

### Establishment of a localized subcutaneously bioluminescent canine DLBCL xenograft model

To establish the bioluminescent xenograft model, a pilot tumor induction study was first conducted with three different cell densities. For that, nine SCID mice were randomly assigned to three different groups, according to the cell density used for inoculation: group I—1×10^6^ cells (n = 3), group II—0.5×10^6^ cells (n = 3) and group III—0.1×10^6^ cells (n = 3). Suspensions of CLBL-1^GFP+Luciferase+^ cells in PBS with matrigel (Corning, NY, USA, Cat # 354248) (1:1) were injected subcutaneously into the dorsal interscapular region to induce tumors. Tumor volume and body weight were measured three times per week. Tumor volume was calculated as (width)^2^ × length from electronic caliper measurements. Tumor endpoints criteria included tumor volume diameter superior to 1.5 cm and/or signs of marked changes in locomotion and posture, difficulties in accessing or ingesting food and drink, weight loss ≥15%, signs of pain (grimace scale ≥1). The tumor load in the mice was also analyzed by weekly bioluminescence imaging (BLI) with IVIS system (Xenogen Corp., Alameda, CA) as described below. After two weeks of tumor development, animals reached a humane endpoint and were sacrificed, necropsy was performed by a veterinary pathologist. Tumor and main organs including the liver, kidney, lung, spleen, and intestine were harvested and formalin-fixed.

### *In Vivo* bioluminescence imaging

*In vivo* bioluminescence imaging (BLI) was conducted on a cryogenically cooled IVIS system (Xenogen Corp., Alameda, CA) using LivingImage acquisition. Prior to BLI imaging, mice received a 150 mg/kg intraperitoneal injection with D-Luciferin (Xenolight, potassium salt). D-Luciferin was purchased from PerkinElmer´s and was dissolved to 15 mg/ml in PBS, filter-sterilized. Fifteen minutes after substrate injection, animals were anesthetized by intraperitoneal injection with a mixture of medetomidine (1 mg/Kg) and ketamine (75 mg/Kg). A photographic image of the animal was taken in the chamber under dim illumination, followed by acquisition and overlay of the pseudocolor image representing the spatial distribution of photon counts produced by active luciferase within the animal. An integration time of 1min with a binning of 100 pixels was used for luminescent image acquisition. Acquired images were analyzed using Living Image Software version 4.5.5 (Xenogen Corp.). Signal intensity was quantified as the sum of all detected photon counts within the region of interest after subtraction of background luminescence measured at the dorsal trunk.

### Histopathological analysis

Tissues, including tumors, were fixed in 10% buffered formalin and embedded in paraffin using a Leica tissue processor. Three μm sections were cut from paraffin blocks and stained with hematoxylin & eosin (H&E). Sections were mounted onto superfrost ultra plus slides (Menzel-Glaser, Braunschweig, DE) for immunohistochemistry.

### Imunohistochemistry analysis

A representative area of each tumor was selected and tissue sections of 3 μm thickness were mounted on glass slides (Superfrost glass slides, Thermo Scientific, Braunschweig, Germany), deparaffinized with xylene and hydrated in a graded ethanol series to distilled water. All protocol steps were carried out using the Novolink Polymer Detection System (Novocastra, Leica Biosystems, Newcastle, UK), according to the manufacturer’s instructions. The antigen retrieval step was performed by microwave treatment (5min at 900 watts plus 15min at 650 watts) in Tris–EDTA buffer (pH 9.0). The system’s Peroxidase Block Solution and Protein Block Solution were used sequentially to block endogenous peroxidase and to prevent unspecific labelling, respectively. Tissue sections were incubated 30min at room temperature with two antibodies: polyclonal rabbit anti-human CD20 (Thermo Fisher Scientific), diluted 1:200, and rabbit polyclonal anti-human CD3 (Dako, Glostrup, Denmark), diluted 1:400. For all antibodies, labelling was developed by incubating the slides with the system´s chromogen, diaminobenzidine (DAB), and hydrogen peroxide as substrate. Nuclear background staining was performed with Gill’s hematoxylin (30sec). Labelling without the primary antibody was used as negative control. Dog lymph node sections were used as positive control.

### Assessment of therapeutic response in the bioluminescent mouse model of canine DLBCL

To validate the bioluminescent canine DLBCL xenograft model for preclinical studies and its potential to investigate the utility of BLI in monitoring response to therapy, a therapeutic study was conducted with panobinostat, a histone deacetylase (HDAC) inhibitor. For this purpose, ten SCID mice were injected subcutaneously into the dorsal region with suspensions of 1 × 10^6^ cells of CLBL-1^GFP+Luciferase+^ cells in PBS with matrigel (1:1) to induce tumors. When tumors reached a minimum volume of 100 mm^3^, mice were randomly assigned to one of the two groups: control group (vehicle only, n = 5) and treatment group (20 mg/kg panobinostat, n = 5). Vehicle (2% DMSO + 48% PEG300 + 2% Tween 80 + ddH_2_O) and treatment dose selection were based on our previous studies [27]. Panobinostat (Selleckchem, Houston, TX, Cat # S1030) stock solutions were prepared at 67 mg/ml in dimethyl sulfoxide (DMSO) (Sigma-Aldrich) and stored at -20°C. Treatment consisted of intraperitoneal injections 5 days per week, over two weeks. Tumor volume and body weight was measured three times per week. Tumor volume was calculated as (width)^2^ × length. Compound activity was determined by tumor growth inhibition (TGI). TGI was determined as the percent change in tumor volume of treated over control animals (%T/C). At the end of the study, all animals were examined using *in vivo* bioluminescence imaging, as described above, and were sacrificed for necropsy examination by a pathologist. Tumor and main organs, including the liver, kidney, lung, spleen and intestine, were collected and formalin-fixed.

### Statistical analysis

All data was expressed as mean ± standard error of mean (SEM). Analysis was performed using Prism 5 (Graphpad Software). All data normality was analyzed using Shapiro-Wilk’s test. For *in vitro* assays, statistical significance of results was determined by One-way ANOVA followed by Tukey Multiple Comparison test to compare individual groups. The distribution of the *in vivo* assays results did not pass the normality test. Therefore, groups were compared using the Mann–Whitney U-test.; p values < 0.05 were considered statistically significant.

## Results

### Generation of a stable CLBL-1^GFP+luciferase+^ cell line

**Fig 1:**
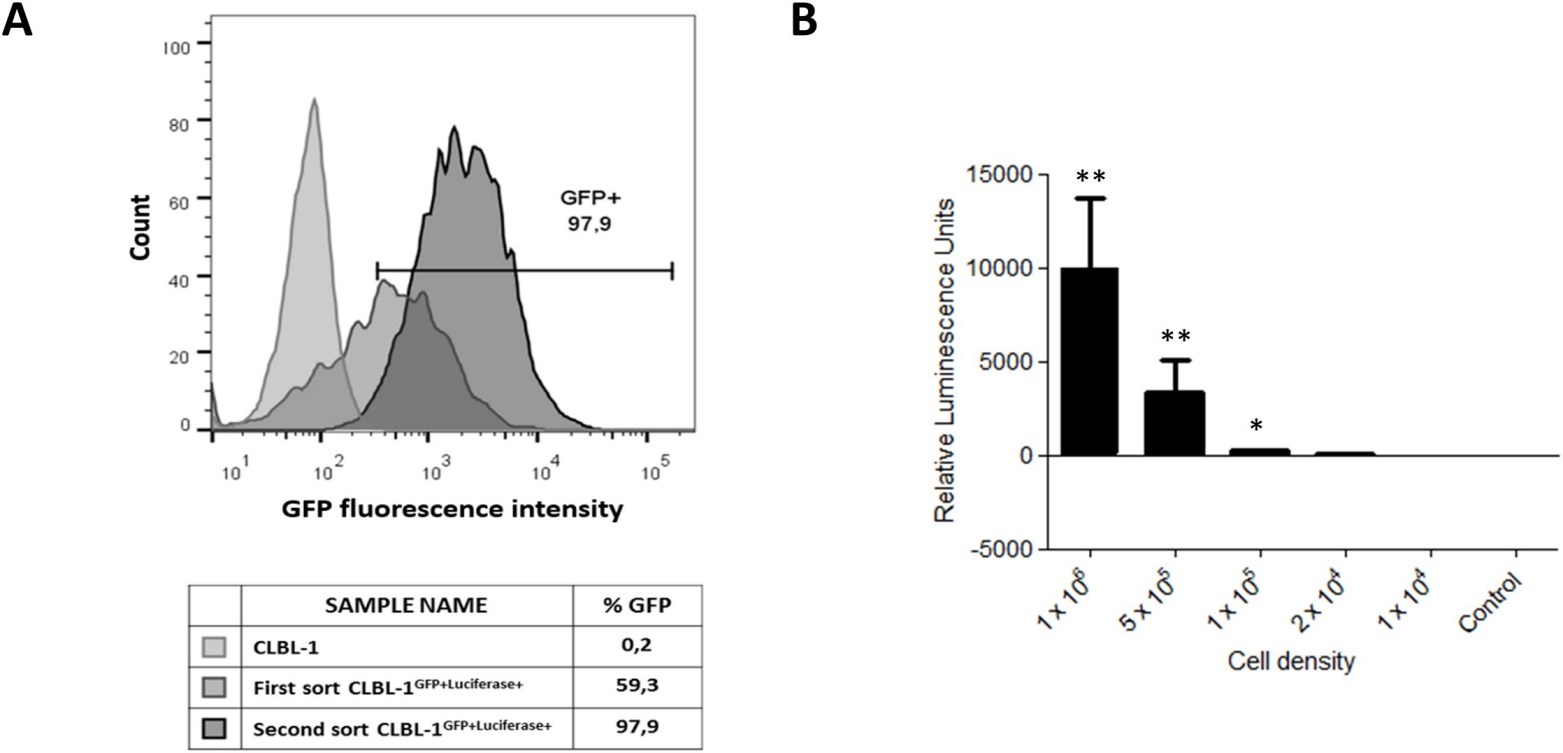
Generation of a stable CLBL-1^GFP+luciferase+^ cell line. (A) Histogram of CLBL-1^GFP+luciferase+^ cell line construction, representing GFP expression analysis after the first and second cell sort. CLBL-1 cells were transduced with lentiviral particles encoding GFP-luciferase reports. After 72h, GFP positive cells were sorted, using FACSAria IIu sorter (BD Biosciences), and cultured in RPMI medium. After 4 weeks in culture, cells were subjected to a second sort to ensure a stable GFP expressing cell line. The phenotype of the stable CLBL-1^GFP+luciferase+^ cell line, following the cell line maintenance for two months, that was used in further assays, was confirmed and is represented by the right-side histogram. (B) Luciferase activity was analyzed in the CLBL-1^GFP+luciferase+^ cell line using the parental cell line as a control (Control—1×10^6^ cells). Indicated cell densities of both cell lines were lysed, incubated with D-luciferin and luminescence was measured. Results are expressed as mean ± SEM. *p < 0.05 and ** *p <* 0.01 from control cells.

The CLBL-1 cell line [23,24] was transduced with a bicistronic lentiviral vector, as described in the material and methods section, to generate a stable canine DLBCL cell line expressing both firefly luciferase and GFP reporters for bioluminescence and fluorescence detection. The CLBL-1 cell line was selected for our study because it is the only canine cell line that faithfully represents diffuse large B-cell lymphoma (DLBCL), reproducibly inducing tumors and preserving its phenotype in the xenotransplantation setting [11,23,24,27]. As shown in Fig 1A, a stable CLBL-1^GFP+luciferase+^ cell line was generated after two cycles of cell sorting. The phenotype of the stable CLBL-1^GFP+luciferase+^ cell line, following the cell line maintenance for two months was analyzed and a 98,4% of GFP cell expression was assessed. As shown in Fig 1B, the luciferase activity of the stable CLBL-1^GFP+luciferase+^ cell line was confirmed using a luciferase assay kit and it was correlated with cell density. In contrast, no luciferase activity was observed for the parental CLBL-1 cells. Importantly, the evaluation of growth patterns through a cell doubling time assay confirmed that the stable CLBL-1^GFP+luciferase+^ cell line exhibited a similar doubling time compared to the CLBL-1 parental cell line (26.45 hour doubling time for CLBL-1^GFP+luciferase+^, versus 26.52 hours for the parental CLBL-1 cell line) (S1 Fig).

### Establishment of a subcutaneously bioluminescent canine DLBCL mouse model

**Fig 2:**
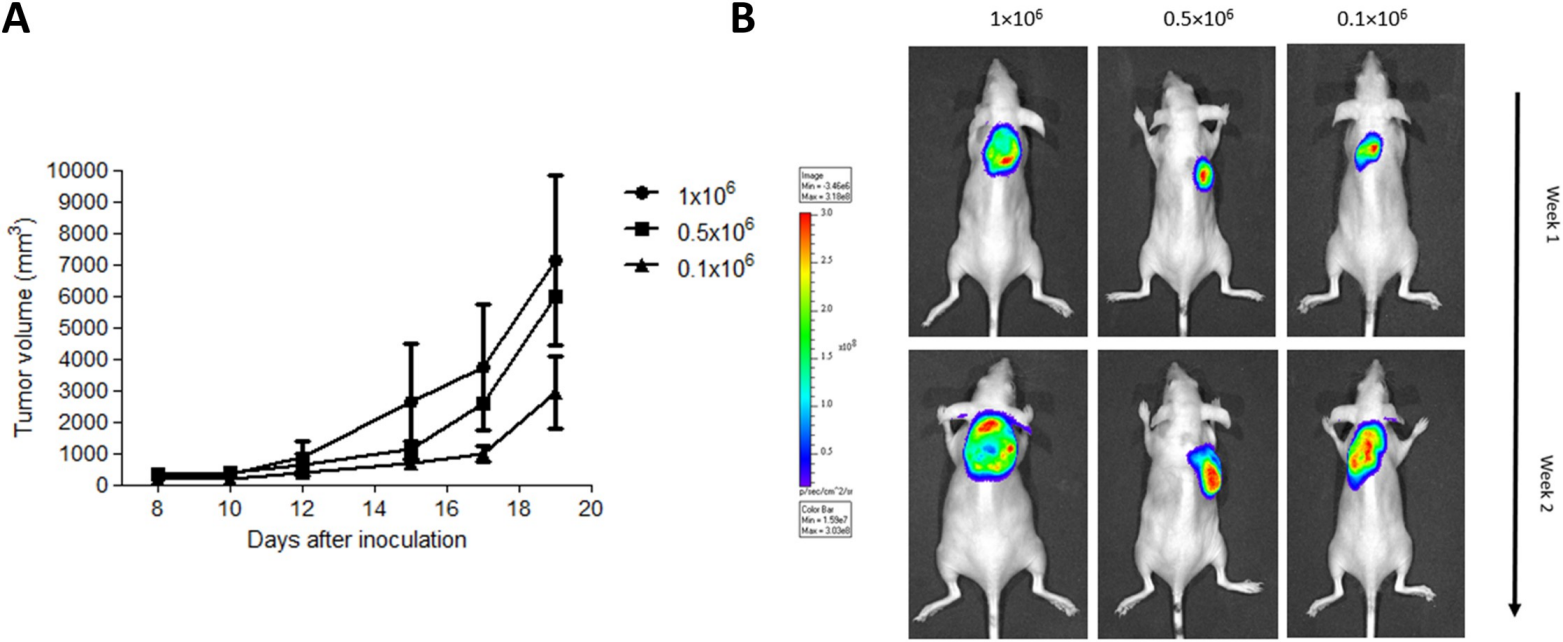
Establishment of a bioluminescent mouse model of canine DLBCL. SOPF/SHO SCID mice (6–8 weeks-old) were injected subcutaneously with CLBL-1^GFP+Luciferase+^ cells using three different cell densities (1×10^6^ cells (n = 3), 0.5×10^6^ cells (n = 3) and 0.1×10^6^ cells (n = 3)) in a matrigel suspension. (A) Tumor volumes were measured three times a week, using a caliper and calculated as (width)^2^ × length (±SEM). There were no significant differences in tumor size between groups. (B) Bioluminescent imaging was performed to monitor tumor development. Prior to BLI imaging, mice received an intraperitoneal injection with D-Luciferin. Fifteen minutes after substrate injection, animals were anesthetized and subjected to *in vivo* imaging. Representative images of bioluminescence imaging at the end of the first and second week are shown.

To develop the subcutaneous bioluminescent canine DLBCL xenograft mouse model a pilot study was first performed with SCID mice inoculated with three different cell densities. For that, mice were randomly assigned to three distinct groups (n = 3) according to the number of cells administered (group I = 1×10^6^ cells, group II = 0.5×10^6^ cells and group III = 0.1×10^6^ cells). Suspensions of CLBL-1^GFP+luciferase+^ cells were inoculated subcutaneously into the dorsum of SCID mice. All xenograft mice, regardless of cell density, presented tumor development at the site of injection eight days after cell inoculation. Importantly, tumors were established with a success rate of 100% (n = 9) (Fig 2A). All tumors were efficiently monitored and quantified by bioluminescence imaging (BLI). No significant differences in tumor growth were observed between groups. As shown in Fig 2B, the BLI signal obtained confirmed tumor induction and allowed for tumor growth monitoring. In addition, it is important to mention that apart from tumor formation, no clinical abnormalities were observed in any of the transplanted mice during the experimental study.

### Characterization of xenograft tumor histopathological features

**Fig 3:**
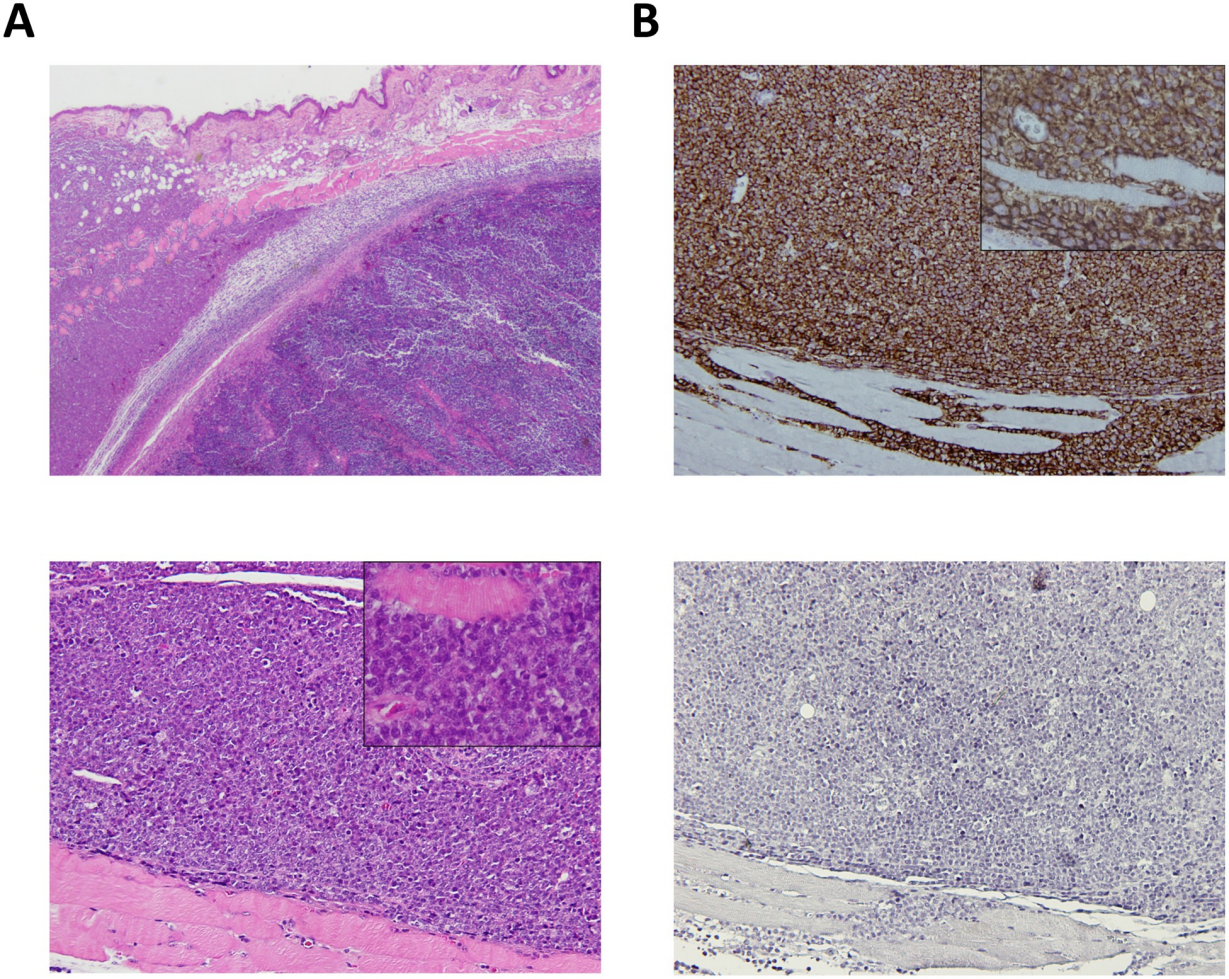
Histopathological characteristics of the CLBL-1^GFP+Luciferase+^ cell line as a xenograft tumor in SOPF/SHO SCID mice. Mouse interscapular region. Xenograft CLBL-1^GFP+Luciferase+^ tumor. (A) Upper panel—Compact infiltration of the dermis, hypodermis, muscle panniculus and skeletal muscle by large lymphoid cells (H&E, 20x). Bottom panel–Magnification of the tumor showing lymphoid cells with indistinct cytoplasmic borders and finely distributed nuclear chromatin and inconspicuous nucleolus. A muscle fiber is surrounded by tumor cells in the insert (H&E, 100x, insert 400x). (B) Upper panel–Immunohistochemistry for B-cells showing positivity in virtually 100% of the tumor cells (anti-CD20 antibody, Gill’s hematoxylin, 100x, insert 400x). Bottom panel–Immunohistochemistry for T-cells, showing that tumor cells were negative for this marker (anti-CD3, Gill’s hematoxylin, 100x).

To assess macroscopic and microscopic characteristics of the bioluminescent canine DLBCL xenograft model, necropsy and histopathological evaluation were performed by a veterinary pathologist. Macroscopically, all xenografts were located in the injection site, the dorsal interscapular region. Tumors were nodular, soft and hemorrhagic and highly adherent to subcutaneous tissue and underlying muscle. No gross macroscopic alterations were identified in the main organs examined. Microscopically, tumors corresponded to compact infiltration of the dermis, hypodermis, muscle panniculus and skeletal muscle by large lymphoid cells with indistinct cytoplasmic borders, finely distributed nuclear chromatin and inconspicuous nucleolus (Fig 3A). There were extensive areas of necrosis and mitotic activity was considered intermediate (six to seven mitosis per high power field). These microscopic features are characteristic of a medium to high grade lymphoma [23]. Immunohistochemistry analysis of the xenograft tumor using CD20 and CD3 labelling was positive for CD20 in virtually 100% of the tumor cells confirming the phenotype of the CLBL-1^GFP+luciferase+^ cell line (Fig 3B). Importantly, all these characteristics were consistent with data obtained for the parental CLBL-1 xenograft model [23,24,27].

### Evaluation of bioluminescence canine DLBCL xenograft model for non-invasive monitoring tumor progression and response to therapy

**Fig 4:**
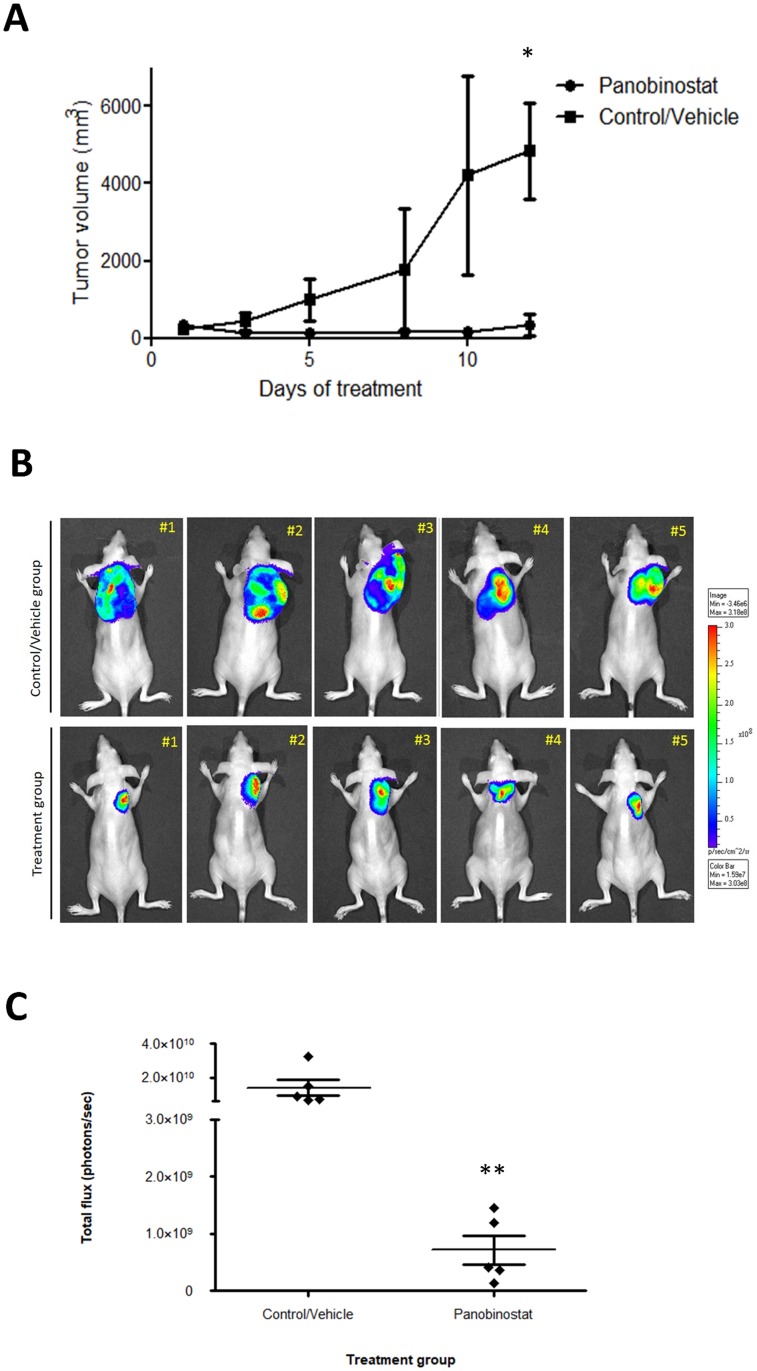
Assessment of therapeutic response of the bioluminescent canine DLBCL xenograft mouse model. SOPF/SHO SCID mice (6–8 weeks-old) were injected subcutaneously with 1 × 10^6^ of CLBL-1^GFP+Luciferase+^ cells. When tumors reached ~100 mm^3^, mice were randomized into two treatment groups: not treated (controls/vehicle only) and panobinostat at 20 mg/kg (n = 5 per group). Mice were treated with intraperitoneal injections for 2 weeks, 5 days per week. (A) The tumor growth curve showed that treatment group had a statistically significant tumor growth inhibition compared to the vehicle group (*p < 0.05). (B) Bioluminescent imaging was performed to monitor therapeutic response. Fifteen minutes after D-Luciferin substrate injection, animals were anesthetized and subjected to *in vivo* imaging. Representative images of bioluminescence imaging at the end of the therapeutic assay are shown. (C) Quantitative analysis of photon counts derived from CLBL-1^GFP+Luciferase+^ xenograft mice between control/vehicle mice and mice receiving panobinostat treatment. The treated mice group presented a significant lower BLI signal, up to 20-fold, compared to untreated control mice; **p < 0.01 when compared to the vehicle control treatment.

Aiming to evaluate the suitability of the established bioluminescent canine DLBCL xenograft model for monitoring tumor progression and therapeutic responsiveness, we conducted an *in vivo* therapeutic study using panobinostat, a HDAC inhibitor. We have recently investigated antitumor properties of HDAC inhibitors for the treatment of canine DLBCL [27]. Among a panel of HDAC inhibitors studied, panobinostat proved to be the most promising compound showing strong *in vitro* and *in vivo* antitumor properties against canine DLBCL. Therefore, panobinostat was selected to test the treatment response in the bioluminescent CLBL-1^GFP+luciferase+^ xenograft model. Considering that in the pilot study all cell densities presented the same engraftment efficiency (100%), the 1×10^6^ cell density was used to establish the xenograft model and the therapeutic study, allowing the further comparison with the xenograft model established using parental CLBL-1 cell line [27]. As expected and consistent with the pilot study, all inoculated mice presented tumors eight days after inoculation. When tumors reached ~100 mm^3^, mice were randomized into two groups: control group (not treated/vehicle, n = 5) and treated group (panobinostat at 20 mg/kg, n = 5). After two weeks of treatment, panobinostat at 20 mg/kg dose inhibited tumor growth by 93.3% when compared to vehicle control treated mice (p < 0.05) (Fig 4A). This tumor growth inhibition was similar to data gathered from the panobinostat efficacy study performed on the xenograft model using the parental CLBL-1 cell line [27]. In addition, besides macroscopic dimensions, xenograft tumors of the panobinostat treated group presented identical histopathological characteristics to the xenograft tumors of the control group (S2 Fig). To validate the bioluminescence model for the detection of a therapeutic response, we quantified the photon signal intensity from BLI and statistically examined the differences between the two groups. As shown in the representative photographs (Fig 4B) and in the BLI measurements (Fig 4C), a significant lower BLI signal, up to 20-fold, was observed in treated mice compared to untreated control mice (p < 0.01). Thus, the BLI signal obtained showed to be extremely suitable for visualization of tumor localization in mice and to monitor the tumor response to the therapeutic molecule.

## Discussion

Diffuse large B-cell lymphoma (DLBCL) is the most common canine aggressive B-cell lymphoma worldwide, sharing similar biological, behavioral, genetic, and molecular characteristics with the human counterpart [6,7,28]. Despite having good response to multiagent chemotherapy, curative treatment remains elusive for most dogs [29]. As such, collaborative efforts are being made to integrate naturally occurring canine lymphoma into novel cancer treatment studies, in order to improve the treatment for dogs diagnosed with lymphoma, while accelerating therapeutic development for human lymphoma [30,31]. However, the integration of canine lymphoma in comparative studies has been limited due in part to the lack of validated, well-characterized and widely disseminated canine lymphoma models for preclinical research [9].

To date, few *in vivo* canine lymphoma models have been described [20–23,32]. This is mainly due to the low number of available well-characterized canine lymphoid cell lines. In fact, canine hematopoietic cell lines have been historically difficult to establish and most cell lines are of T-cell origin [9,33]. Therefore, the majority of canine lymphoma xenograft murine models described until now represent T-cell lymphoid malignancies [20–22]. Notably, only four of the available cell lines are reportedly of B-cell origin, including the GL-1 cell line derived from a dog with B-cell acute lymphoblastic leukemia [34]; the 17–71, a B-cell cell line not initially phenotyped and that does not express typical B-cell lymphoma markers [35]; 3132, a cell line that probably is not of B-cell origin despite initial reports of surface immunoglobulin [36] and CLBL-1, the only available cell line that has been well-characterized both *in vitro* and *in vivo* [11,23,24,27,37]. As a matter of fact, CLBL-1 appears to be the only exclusive cell line that faithfully represents DLBCL, reproducibly inducing tumors and preserving its phenotype in the xenotransplantation setting [23,24]. Primary canine DLBCL xenografts have also been described and are a possible alternative approach, however, these tumors only form when implanted intraperitoneally into conditioned NOD *scid* gamma (NSG) mice and their variability in growth makes therapeutic evaluation challenging [11,32].

The CLBL-1 cell line tumorigenicity, genomic stability, histological and morphological properties were initially reported on a xenograft murine model [23]. For that purpose, CLBL-1 cell line was subcutaneously injected in the right and left flank of Rag2−/−γc−/− mice. This model was highly tumorigenic, and all mice demonstrated liver, spleen, bone marrow, ovaries and uterus lymphoma involvement. This was the first study demonstrating that CLBL-1 canine lymphoma cell line develops multicentric lymphoma as observed in canine patients, making it a highly stable tool for B-cell lymphoma research in veterinary and human medicine. Nevertheless, it revealed certain shortcomings related to heterogeneous clinical presentation and inability to monitor disease progression through non-invasive methods [23]. A similar *in vivo* study with CLBL-1 in murine xenograft model has also been reported by Weiskopf *et al*. to study the synergy of the antitumor activity of blocking CD47 and anti-CD20 immunotherapy. This work established xenograft models of disseminated, intra-abdominal and subcutaneous disease into NSG mice, paving the way for the development of bioluminescent canine B-cell lymphoma mouse models. However, these models were not established using a stably expressed form. Furthermore, many questions remained to be answered regarding tumor engraftment efficiency, reproducibility and BLI monitoring [11].

To overcome these limitations, we aimed at establishing and characterizing a localized subcutaneous bioluminescent xenograft mouse model of canine DLBCL, which would easily allow monitoring of tumor progression and treatment response in preclinical studies. For this purpose, we established a SCID xenograft model of canine DLBCL by subcutaneously implanting CLBL-1^GFP+Luciferase+^ cells. The development of a stable cell line of CLBL-1 expressing luciferase and GFP, allows monitoring and quantifying the disease progression noninvasively. Currently, bioluminescent imaging (BLI) is one of the most widely used techniques to track target cells *in vivo*, especially hematopoietic cell lines that disseminate widely in their hosts as xenografts [38]. This technique relies on the fluorescent signal produced by the chemical reaction between the luciferase and its substrate (D-luciferin), as such it is highly specific and sensitive, allowing to visualize, quantify and monitor in real-time the tumor development [39]. CLBL-1^GFP+ Luciferase+^ cell line implanted in SCID mice induced highly aggressive tumors, with rapid tumor growth that requires close monitoring to avoid tumor burden. Three different cell densities for tumor establishment were tested (0.1×10^6^, 0.5×10^6^ and 1×10^6^ cells) and all presented homogeneous tumor development within eight days after injection, with a 100% engraftment success rate. There were no significant differences in tumor growth curve between different cell density groups.

Histological and immunohistochemical analysis revealed that xenograft tumors retained similar histological characteristics and B-cell and T-cell markers expression, compared to original CLBL-1 cell line xenografts [23,24]. Finally, the CLBL-1^GFP+Luciferase+^ model, comparable to the parental CLBL-1 model, demonstrated a high consistency in disease progression with tumor onset occurring after 8 days of inoculation in all animals, providing an intervention window of two weeks that allows the rapid screening of a plethora of therapeutic molecules.

In order to confirm whether the bioluminescent canine DLBCL xenograft model could be a reliable preclinical tool for drug investigation, we conducted a therapeutic study. We have recently investigated antitumor properties of a panel of seven HDAC inhibitors for the treatment of canine DLBCL. Amongst all HDAC inhibitors studied, panobinostat proved to be the most promising compound and was selected for further *in vitro* and *in vivo* investigation. This potent HDACi demonstrated strong antitumor properties against a CLBL-1 xenograft canine tumor growth, as it efficiently inhibited tumor growth [27]. As such, panobinostat was selected to test the treatment response in our established bioluminescent model. The results presented herein, demonstrated that treatment with panobinostat (20mg/kg) efficiently inhibited tumor growth and consequently reduced the BLI signals, up to 20-fold, when compared with the control mice. Thus, the BLI measurements obtained with the established bioluminescence xenograft model were extremely suitable for visualization of the tumor localization in the mice, but also highly useful for the quantitative detection of the tumor load and response to therapy.

In conclusion, in this study we established and characterized a novel localized subcutaneous bioluminescent canine DLBCL xenograft model that offers high engraftment efficiency, preservation of relevant tumor features and reproducible tumor growth. This model established with CLBL-1^GFP+Luciferase+^ cells can be therefore efficiently used to monitor non-invasively and quantitatively the outgrowth of canine DLBLC, and be a valuable preclinical tool for veterinary applications, while contributing to comparative oncology.

## Supporting information

S1 Fig Growth curves of the CLBL-1^GFP+luciferase+^ cell line versus the parental CLBL-1 cell line. The cells were grown for 5 days in the regular cell culture conditions and as described in the material and methods section. The total numbers of cells over time are plotted in a logarithmic scale. Both cell lines showed similar growth patterns and doubling times.
(TIF)

S2 Fig Histopathological characteristics of the CLBL-1^GFP+Luciferase+^ xenograft tumor in SOPF/SHO SCID mice after panobinostat treatment. Mouse interscapular region. Xenograft CLBL-1^GFP+Luciferase+^ tumor. Left—Compact infiltration of large lymphoid cells exclusively of the hypodermis (H&E, 20×). Right—Magnification of the large lymphoid cells part of which are necrotic with nuclei in karyorrhexis (H&E, 400×).
(TIF)
