# Supplementary figures and images for: Establishment of a bioluminescent canine B-cell lymphoma xenograft model for monitoring tumor progression and treatment response in preclinical studies
Source: PLoS One. 2018 Dec 28;13(12):e0208147. doi: 10.1371/journal.pone.0208147 (PMC6310248; doi:10.1371/journal.pone.0208147)

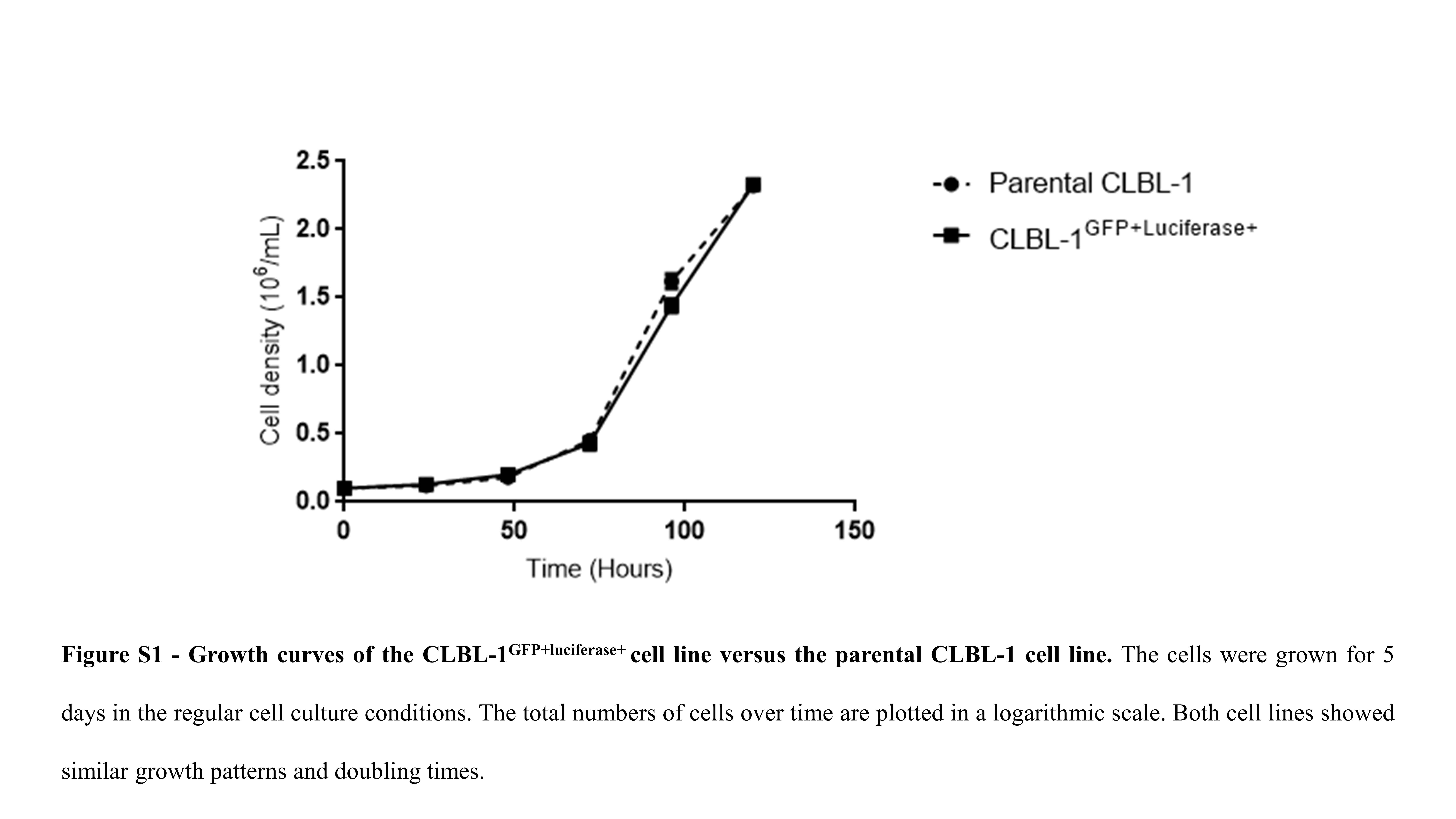

Supplement: S1 Fig — The cells were grown for 5 days in the regular cell culture conditions and as described in the material and methods section. The total numbers of cells over time are plotted in a logarithmic scale. Both cell lines showed similar growth patterns and doubling times. (TIF) [file pone.0208147.s001.tif]

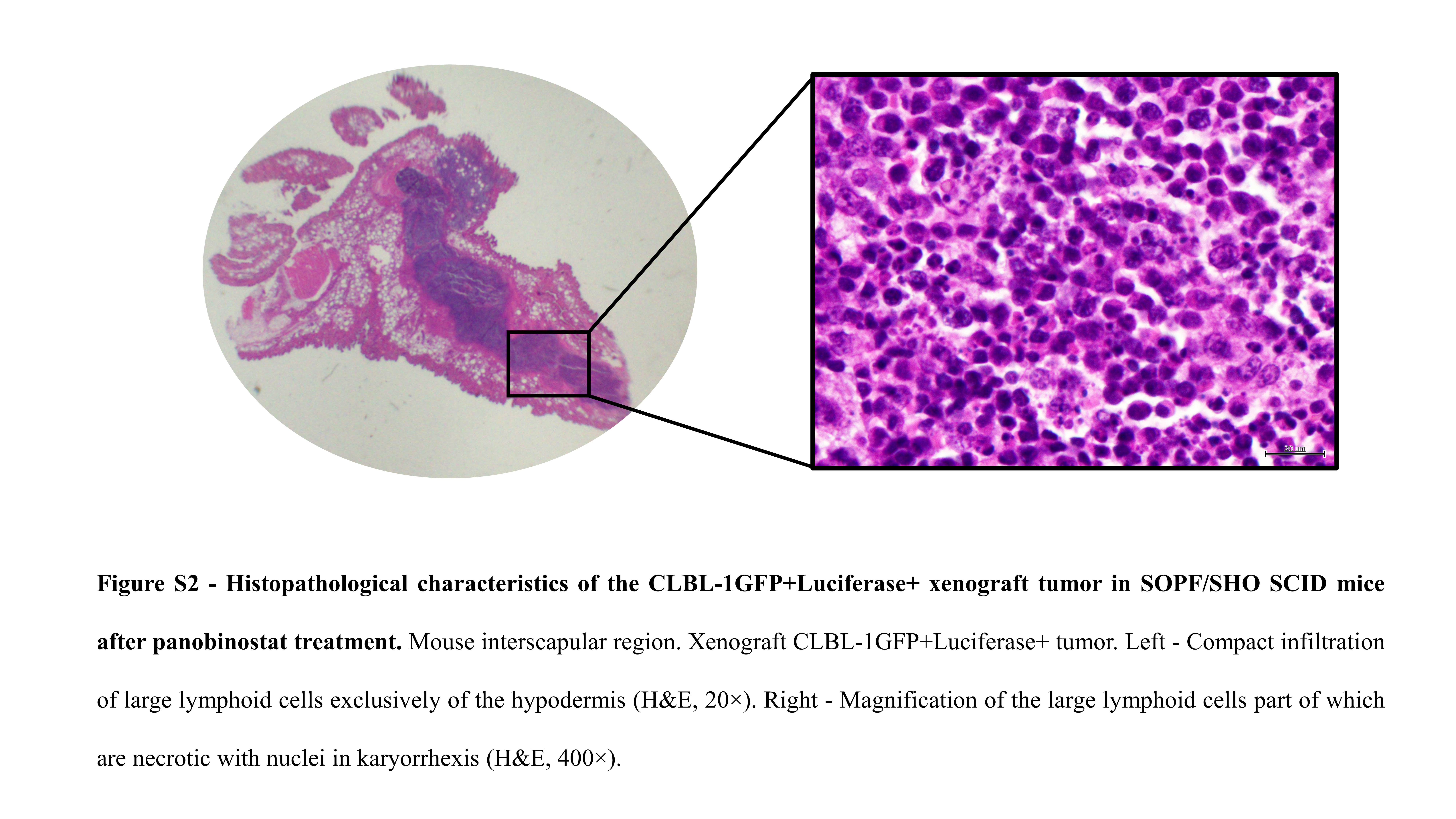

Supplement: S2 Fig — Mouse interscapular region. Xenograft CLBL-1GFP+Luciferase+ tumor. Left—Compact infiltration of large lymphoid cells exclusively of the hypodermis (H&E, 20×). Right—Magnification of the large lymphoid cells part of which are necrotic with nuclei in karyorrhexis (H&E, 400×). (TIF) [file pone.0208147.s002.tif]
